# Persistent Persister Misperceptions

**DOI:** 10.3389/fmicb.2016.02134

**Published:** 2016-12-27

**Authors:** Jun-Seob Kim, Thomas K. Wood

**Affiliations:** Department of Chemical Engineering, Pennsylvania State University, University ParkPA, USA

**Keywords:** persister cells, antimicrobial tolerance

## Abstract

Persister cells survive antibiotic treatment due to their lack of metabolism, rather than through genetic change, as shown via four seminal experiments conducted by the discoverers of the phenotype (Hobby et al., 1942; Bigger, 1944). Unfortunately, over seven decades of persister cell research, the literature has been populated by misperceptions that do not withstand scrutiny. This opinion piece examines some of those misunderstandings in the literature with the hope that by shining some light on these inaccuracies, the field may be advanced and subsequent manuscripts may be reviewed more critically.

## Reveiw

Persister cells survive antibiotic treatments by reducing their metabolism; i.e., rather than by mounting an active response to the antibiotic stress, such as activating eﬄux pumps and rather than undergoing mutation, they survive antibiotics by becoming dormant. By dormant, the experimental evidence indicates persister cells are non-growing (Hobby et al., 1942; Bigger, 1944) and lack transcription, translation, and proton-motive force (Kwan et al., 2013). Furthermore, given the widespread use of the synonym “tolerant” and its contrived distinction with persistence (Brauner et al., 2016), perhaps a working definition is best: persister cells are those cells that remain after the actively respiring cells are vanquished by antibiotics (usually seen as an unchanging or slowly decreasing concentration of viable cells). In the following paragraphs, each misperception about persistence is shown in bold followed by a discussion which examines it.

### Misperception 1: Persister Cells Are Not Dormant

When Hobby et al. (1942) first described persister cells, they showed that penicillin was ineffective against non-growing (cold) *Staphylococcus aureus* cells; i.e., persister cells are dormant. Bigger (Bigger, 1944) then confirmed that persister cells are dormant by showing (i) lowering the culture temperature to stop growth generates more persisters to penicillin, (ii) stopping growth by removing nutrients generates more persisters, and (iii) ceasing growth by adding boric acid at bacteriostatic concentrations generates more persisters.

Later research corroborated that persister cells are metabolically inactive; for example, Shah et al. (2006) found that metabolically inactive cells were more tolerant to the fluoroquinolone ofloxacin (although the sorting process itself in non-nutritive medium may have introduced artifacts), and Kwan et al. (2013) found that cells lacking protein synthesis, via pre-treatment with rifampin to stop transcription, with tetracycline to stop translation or with carbonyl cyanide *m*-chlorophenylhydrazone to halt ATP production, become persister cells. These three pre-treatments convert an initial population of 0.01% persisters to up to approximately 80% persisters (a 10,000-fold increase in persister cells). Hence, persister cells are predominantly dormant, although there may be a gradient in their metabolic activity, as they clearly must reduce their metabolism.

In contrast, Orman and Brynildsen (2013) misinterpreted a set of fluorescence-activated cell sorting (FACS) experiments to claim *E. coli* persister cells were not necessarily dormant when they likely inadvertently carried over high numbers of dormant persister cells into their FACS experiments (Wood et al., 2013). Furthermore, the statistical errors in their technique were greater than the number of cells they used to make their conclusions, and the bulk of their data indicated persister cells are inactive. Paradoxically, the same group now claims, using more complicated FACS approaches, that persister cells are metabolically inactive as they write, “we used Persister-FACSeq on a library of reporters to study gene expression distributions in non-growing *Escherichia coli*, and found that persistence to ofloxacin is inversely correlated with the capacity of non-growing cells to synthesize protein” (Henry and Brynildsen, 2016).

Other groups continue to claim persister cells are metabolically active, but there is usually a flaw in their procedures that nullifies their conclusion. For example, one common mistake is to convert persister cells into actively growing cells by providing a carbon source. This mistake was made by Orman and Brynildsen (2013) since the authors collected their *E. coli* persister cells by FACS but then mixed them into rich medium, which likely activated the formerly persister cells. Similarly, this mistake led to the incorrect conclusion that glycolysis intermediates “potentiate” the killing of persister cells by aminoglycoside antibiotics when in fact the authors merely woke the persister cells with nutrients and were studying actively growing cells, not persister cells, as evidenced by their supplemental data which showed the cells they were claiming to be persisters were only growing (Allison et al., 2011).

Pu et al. (2016) concluded that persister cells utilize the TolC eﬄux pump, may also have been studying actively growing cells since it is well-known that active cells utilize eﬄux pumps in an effort to survive antibiotic treatments. This speculation is based on the fact that the authors indicated in their supplemental material that they treated with antibiotics then washed with “M9 minimal medium” which presumably has glucose and then incubated with the fluorescent antibiotic BOCILLIN FL, so persister cells would have been converted to actively growing cells by the M9 minimal medium due to the presence of a carbon source. Furthermore, their conclusion that persister cells have greater eﬄux would be incorrect if the persister cells have less penicillin binding proteins (PBPs) due to their reduced protein production; i.e., the reduced fluorescence that was used to indicate more active eﬄux may be due to an artifact of lower PBPs in the persister cells since BOCILLIN FL binds PBPs.

For the work of Wakamoto et al. (2013), there is also an artifact. In this work, cells that were not killed by the prodrug isoniazid were indeed metabolically active. However, since isoniazid must be activated by catalase KatG, the metabolically active cells tolerant of isoniazid simply had low levels of KatG. Hence; the metabolic activity observed for cells surviving isoniazid cannot be concluded to be related to metabolic activity in persister cells (Wood et al., 2013).

### Misperception 2: Guanosine Tetraphosphate (ppGpp) Activates Lon Protease to Increase Persistence

In regard to a mechanism of persister cell formation, it has been proposed that the ppGpp that mediates the stringent response activates Lon protease through polyphosphate, then Lon inactivates antitoxins of toxin/antitoxin (TA) systems, freeing the toxin to reduce metabolism (Maisonneuve et al., 2013). Since ppGpp was first linked to persistence in 2003 via the HipA toxin (Korch et al., 2003), the only novel part of this model is the connection of ppGpp to Lon protease via polyphosphate. However, one of the many flaws in this model is that at least *in vitro*, both ppGpp and polyphosphate deactivate Lon protease, rather than activate it (Osbourne et al., 2014). Furthermore, *lon* deletions in other labs have had no effect on persistence with aminoglycosides or β-lactams (Shan et al., 2015; Chowdhury et al., 2016a). Similarly, if the enzyme that produces polyphosphate, PpK, is inactivated, the number of persisters are not altered significantly (Shan et al., 2015). Critically, in cells that no longer produce ppGpp, persister cells are still generated (Chowdhury et al., 2016a) so there are clearly other methods by which the cell becomes persistent. Also, this model does not hold for many type II TA systems since the authors subsequently published that it was not valid for toxins RelE, MazF, and YafO (Germain et al., 2015) and others showed it is not valid for the YoeB/YefM TA system (Ramisetty et al., 2016). Therefore, this flawed model appears to hold for the HipA toxin but a highly flawed system (Germain et al., 2015) was used to show its relevance for this TA system: both the flawed strain in which 10 type II TA systems were deleted (“delta 10”; see Misperception 3 below) and a non-toxic toxin was used (see Misperception 5 below). Additionally, this model is not relevant for type I (Verstraeten et al., 2015) or type III TA systems.

### Misperception 3: It Is Necessary to Delete Multiple TA Systems to See an Effect on Persistence

This misperception is frequently propagated, even by authors who have proved it false. Critically, by deleting a single TA locus, several authors have shown that persistence is reduced; for example, a reduction in persistence upon deleting a single TA locus has been shown for *mqsR* and *mqsRA* (Kim and Wood, 2010; Luidalepp et al., 2011), *tisAB/istR* (Dörr et al., 2010), and *yafQ* (Harrison et al., 2009). Therefore, it is not necessary to reduce several TA loci simultaneously to see an impact on persistence as has been claimed (Maisonneuve et al., 2011). Inexplicably, the laboratory that claimed (incorrectly) to report the first single deletion that reduces persistence (Dörr et al., 2010), who wrote “Here, we show that a knockout of a particular SOS-TA locus, *tisAB/istR*, had a sharply decreased level of persisters tolerant to ciprofloxacin,” now claim (Conlon et al., 2016) that multiple TA systems must be made to reduce persistence: “Although deletion of individual interferases has no phenotype, a knockout of ten TAs produced a decrease in persisters in both a growing culture and in the stationary phase.”

Since toxins of TA systems have different targets, indubitably, deletions of different TA systems should affect persistence to a different degree. Hence, it may be possible to delete some TA systems and see no effect on persistence but it is incorrect to indicate that it is necessary to delete more than one TA system to see an effect on persistence. Part of this misperception may be related to a failure to recognize that TA systems have other roles in different strains, such a phage inhibition (Pecota and Wood, 1996). Hence, not all TA systems are necessarily related to persistence.

### Misperception 4: The *E. coli* Delta 10 Strain Is a Good Tool for Studying Persister Cells

At a time when it was already known that *E. coli* contains many more than 10 TA systems, a report was made about the impact of these 10 TA systems on persistence as well as the role of Lon protease in persistence (Maisonneuve et al., 2011); in this manuscript, the authors deleted 10 TA systems to create a “delta 10” strain. Since *E. coli* has at least 39 TA systems (Tan et al., 2011; Yamaguchi et al., 2011; Wang et al., 2012; Guo et al., 2014), if only 10 of these are deleted, one clearly cannot claim much with confidence in regard to the effect of TA systems on cell physiology since at least 29 other TA systems that may potentially affect the cell. Furthermore, it was announced by the group that constructed that delta 10 strain at the 2015 American Society for Microbiology Meeting in New Orleans that the delta 10 strain contains mutations beyond those related to the TA systems that were deleted; these problems in its construction also invalidate its use. So use of the delta 10 strain suffers from two major flaws: there are at least 29 other functional TA systems in *E. coli* and the delta 10 strain has mutations beyond the deletion of the 10 TA systems that make a comparison to the wild-type invalid.

### Misperception 5: The Non-toxic HipA7 Variant Is a Suitable Tool for Persister Research

Although the link between TA systems and persister cells was first established 33 years ago (in 1983) through the HipA/HipB TA system through the gain of function *hipA7* mutation that increases persistence (Moyed and Bertrand, 1983), there are problems with this system. Notably, the HipA7 variant (containing the amino acid substitutions G22S and D291A) is not toxic (Korch et al., 2003). Therefore, the many persister-related experiments using this variant are not studying an active toxin and conclusions from these studies may be related to persistence but they are not necessarily related to TA systems. For example, *hipA7* mutations have been found in antibiotic-tolerant *E. coli* strains that cause urinary tract infections; yet, activity of the HipA toxin variant after the G22S substitution was not shown (Schumacher et al., 2015). One group has even claimed recently that “Thus, slow growth *per se* does not induce persistence in the absence of TA-encoded toxins”; yet, their methods include the use of both this non-toxic allele (*hipA7*) and the flawed delta 10 host (Germain et al., 2015), so their results are unreliable. Therefore, given the artifacts associated with the *hipA7* strain, the persister field would be better served by using other models in which an active toxin of a TA system is utilized.

### Misperception 6: Persister Cells Are Generated Primarily Spontaneously

The single cell experiments showing stochastic or spontaneous generation of persister cells were based on the *hipA7* strain (Balaban et al., 2004) that does not produce an active toxin (see Misperception 5 above) so the relevance of stochasticity and persistence is questionable. This paper also showed that persister cells were continuously generated in the exponential phase using this suspect strain (Balaban et al., 2004), a result that has been refuted by several groups who showed persister cells are absent from exponentially growing cells, if one is careful not to carry over persisters in the inoculum (Keren et al., 2004; Orman and Brynildsen, 2013). It also does not make sense physiologically to make persister cells spontaneously during times of an abundance of nutrients.

Kwan et al. (2013) demonstrated that pre-treatment with the antibiotics such as rifampicin and tetracycline converts nearly all the *E. coli* cells to the persister state; hence, persister cells go from a small subpopulation to being the main phenotype. These results were corroborated by Van den Bergh et al. (2016) who also demonstrated that pre-treatment with antibiotics converts cells into the persister state. Others have also shown that environmental factors influence the persister state by using the antibiotic ciprofloxacin (Dörr et al., 2010), the interkingdom signal indole (Vega et al., 2012; Hu et al., 2015; Kwan et al., 2015b), and *Pseudomonas aeruginosa* quorum sensing molecules (Möker et al., 2010). Hence, although persister cells may arise spontaneously in small numbers (Balaban et al., 2004), the more germane physiological origin is probably related to the response of cells to stress.

### Misperception 7: Few Methods Exist to Kill Persister Cells

This idea is false as there are many methods now that are effective against persister cells (Wood, 2016). Even in 2016, it has been stated that “Currently, few methods exist to kill persisters, and none are yet clinically developed” (Henry and Brynildsen, 2016). This statement is at best misleading since; for example, mitomycin C (Kwan et al., 2015a; Cruz-Muñiz et al., 2016) and cisplatin (Chowdhury et al., 2016b) have been found to kill persister cells very effectively (by crosslinking DNA while cells sleep). Although both compounds have been approved for use clinically for cancer treatments, they have not been approved for treating bacterial infections.

### Misperception 8: cAMP Increases Persistence

Since cell populations change by orders of magnitude with antimicrobials, many erroneous results have been published based on relatively small changes in persister cell populations. For example, the Brynildsen group reported that exogenous (8 mM) cAMP increases persistence by 20-fold (a relatively modest change in the persister field; Amato et al., 2013). In contrast, others have found that reducing internal cAMP concentrations via three complimentary methods has the opposite effect: (i) degrading cAMP with the cAMP-specific phosphodiesterase CpdA increases persistence 235-fold and an immunoassay confirmed cAMP levels were reduced by 323-fold, (ii) inactivating the single adenylate cyclase that forms cAMP via the *cyaA* mutation increases persistence 19-fold, and (iii) degrading cAMP via non-specific phosphatase DosP increases persistence 4200-fold (Kwan et al., 2015b). Hence, for relatively small changes in persistence (20-fold or less), multiple lines of evidence are required before conclusions may be made with confidence.

### Misperception 9: It Is Difficult to Isolate Persister Cells as an Abundant Phenotype

It has been argued that there is “an inability to isolate persisters from other more abundant cell types” (Henry and Brynildsen, 2016). This statement is highly inaccurate since one may easily isolate an *E. coli* population that is up to 80% persister cells by pre-treating exponentially growing cells with rifampicin, chloramphenicol, or carbonyl cyanide *m*-chlorophenylhydrazone (Kwan et al., 2013). Furthermore, by treating exponentially growing cells via acid or hydrogen peroxide, nearly 100% of the cells will be persistent (Hong et al., 2012). To argue that these persister cells created by pre-treatment are somehow not “bona fide” (Henry and Brynildsen, 2016) and different from persister cells formed after stress, such as the nutrient stress as a result of growth, is disingenuous. Also, heterologous production of any potent toxin or toxic protein makes persister cells at high percentages (Hong et al., 2012; Chowdhury et al., 2016a). More simply, allowing *E. coli* to reach the stationary phase creates a cell population where almost all the cells are tolerant to β lactams (Luidalepp et al., 2011).

### Misperception 10: There Are Difficulties in Obtaining the Metabolome or Transcriptome of Persister Cells

It has also argued in 2016 that there are “difficulties associated with segregating persisters from other cell types have prevented a bona fide transcriptome or metabolome of native persisters to be obtained” (Henry and Brynildsen, 2016) and “Without methods to provide high purity samples, high-throughput (HT) techniques (e.g., RNA sequencing) cannot be employed to provide knowledge of naturally occurring persisters” (Henry and Brynildsen, 2016). These recent claims are inappropriate given that not only has an uniform population of persister cells been created by generating a more toxic toxin of a TA system, but then transcriptomics was used to show that cells that were less fit are more likely to be persisters (Hong et al., 2012). Furthermore, given that persister cells are metabolically inactive, one would expect that the transcriptome of persister cells would not be particularly informative since little (if any) transcription should occur.

### Misperception 11: Interspecies and Interkingdom Signal Indole Increases Persistence

The claim by Vega et al. (2012) that indole increases persistence is erroneous (Hu et al., 2015; Kwan et al., 2015b). Indole is an important signal since it is produced by at least 27 different genera that produce tryptophanase (TnaA) which converts tryptophan into indole (Lee et al., 2007b). Furthermore, indole is one of two quorum-sensing signals (Lee et al., 2007a) in *E. coli*: indole is the primary signal at low temperatures and autoinducer-2 is the primary signal at higher temperatures in the gastrointestinal tract (Lee et al., 2008). Indole is also an interspecies signal that decreases pathogenicity (Lee et al., 2009; Chu et al., 2012; Hidalgo-Romano et al., 2014), alters biofilm formation of neighboring cells (Lee et al., 2007b); tightens epithelial cell junctions to reduce infection (Bansal et al., 2010; Shimada et al., 2013), and increases antibiotic resistance by activating eﬄux pumps (Hirakawa et al., 2005; Kobayashi et al., 2006).

By investigating two different systems, the YafQ/DinJ TA system and cyclic diguanylate (c-di-GMP) signaling, it has been determined that indole instead reduces persistence. The *E. coli dinJ-yafQ* TA locus is induced in persister cells (Keren et al., 2004), and toxin YafQ increases persister cells with multiple antibiotics including ampicillin (1000-fold) and ciprofloxacin (43-fold; Hu et al., 2015). The mechanism for this increase in persistence is the reduction of TnaA levels since RNase YafQ cleaves TnaA mRNA (which has 16 putative YafQ cleavage sites; Hu et al., 2015). Adding indole exogenously also reduces persistence up to 52-fold (Hu et al., 2015).

The second set of results showing indole reduces persistence was obtained by studying ci-di-GMP intercellular signaling and finding production of DosP (direct oxygen sensing phosphodiesterase) increases persistence by over a thousand fold (Kwan et al., 2015b). The mechanism of increasing persistence by DosP is through its degradation of another cyclic nucleotide signal, cyclic adenosine monophosphate (cAMP), which results in lower indole levels due to reduced TnaA activity (Kwan et al., 2015b).

Furthermore, substituted indoles reduce persistence (Lee et al., 2016). By testing 36 diverse indole derivatives for the ability to inhibit persister cells of Gram-negative *E. coli* and Gram-positive *S. aureus*, 5-iodoindole, 4-fluoroindole, 7-chloroindole, and 7-bromoindole were identified that eradicate persister cell formation by both bacteria. 5-Iodoindole also diminished the virulence of *S. aureus*. Hence, it is more likely that indole reduces persistence.

### Misperception 12: There Are No Persister Mechanisms

It has been argued recently in 2016 that “the mechanisms that allow one non-growing, stationary-phase cell to become a persister while approximately 90–99 of its genetically identical kin remain sensitive to antibiotic have yet to be determined” (Henry and Brynildsen, 2016). This statement is false, since, as outlined above, transcriptomics have been used to determine that cells that are less fit to deal with antibiotic stress more readily become persister cells (Hong et al., 2012), since the mechanisms by which indole decreases persistence have been determined (Hu et al., 2015; Kwan et al., 2015b), and since the relationship between the toxin HipA, ppGpp, and persisters has been established (Korch et al., 2003).

## Conclusion

On the surface, it would seem that the study of persister cells should be straight-forward extension of studies on antimicrobials; the reality has been far different in that insights into the mechanisms by which persister cells arise (e.g., indole, TA systems and ppGpp, higher persistence in less fit cells) has been difficult since no one protein is responsible (Chowdhury et al., 2016a; Kaldalu et al., 2016). For example, many studies using transposon-sequencing (Shan et al., 2015), protein expression (Spoering et al., 2006), and gene knockouts (Hu and Coates, 2005; Hansen et al., 2008) have failed to produce a coherent mechanism for persister cell formation. One complicating factor is that relatively large changes in persister cell concentrations must occur for a link to be made conclusively to a phenotype; i.e., changes of fivefold or less are probably meaningless when cell populations change by orders of magnitude (e.g., from 10^9^ to 10^4^ cells/mL). Also, errors in technique abound with many authors waking the persister cells with fresh medium; this leads to the phenotypes of active cells being attributed to persister cells. Another complicating factor is that the field has been side-tracked by concepts such as “potentiation,” stochastic/spontaneous generation (and the associated plethora of bet-hedging claims), and metabolic activity as well as side-tracked by the use of *E. coli* strains such as *hipA7* and delta 10. Clearly the HipA toxin was important for establishing the link between TA systems and persister cells and for elucidating the role of ppGpp, but far better systems exist now for studying persisters; i.e., ones with active toxins. Given that microbial infections are the leading cause of death worldwide (Rasko and Sperandio, 2010) and that persister cells are indubitably a cause of recurring infections (Fauvart et al., 2011), discerning more details for the mechanism of persister cell formation is an essential area of research, and one which should be approached with fewer misperceptions.

## Author Contributions

TW conceived and wrote the manuscript. J-SK helped write the manuscript.

## Conflict of Interest Statement

The authors declare that the research was conducted in the absence of any commercial or financial relationships that could be construed as a potential conflict of interest.
